# Lineage-Selective Disturbance of Early Human Hematopoietic Progenitor Cell Differentiation by the Commonly Used Plasticizer Di-2-ethylhexyl Phthalate via Reactive Oxygen Species: Fatty Acid Oxidation Makes the Difference

**DOI:** 10.3390/cells10102703

**Published:** 2021-10-09

**Authors:** Lars Kaiser, Isabel Quint, René Csuk, Manfred Jung, Hans-Peter Deigner

**Affiliations:** 1 Institute of Precision Medicine, Medical and Life Sciences Faculty, Furtwangen University, Jakob-Kienzle-Straße 17, 78054 Villingen-Schwenningen, Germany; kal@hs-furtwangen.de (L.K.); qui@hs-furtwangen.de (I.Q.); 2 Institute of Pharmaceutical Sciences, University of Freiburg, Albertstraße 25, 79104 Freiburg im Breisgau, Germany; manfred.jung@pharmazie.uni-freiburg.de; 3 Department of Organic Chemistry, Martin-Luther-University Halle-Wittenberg, Kurt-Mothes-Str. 2, 06120 Halle (Saale), Germany; rene.csuk@chemie.uni-halle.de; 4 CIBSS—Centre for Integrative Biological Signalling Studies, University of Freiburg, 79104 Freiburg im Breisgau, Germany; 5 Fraunhofer Institute IZI, Leipzig, EXIM Department, Schillingallee 68, 18057 Rostock, Germany; 6 Associated Member of Faculty of Science, Tuebingen University, Auf der Morgenstelle 8, 72076 Tübingen, Germany

**Keywords:** endocrine-disrupting compounds, fatty acid oxidation, reactive oxygen species, ROS quenching, hematopoietic stem and progenitor cells, di-2-ethylhexyl phthalate, mono-2-ethylhexyl phthalate, hematopoiesis, hematotoxicity

## Abstract

Exposure to ubiquitous endocrine-disrupting chemicals (EDCs) is a major public health concern. We analyzed the physiological impact of the EDC, di-2-ethylhexyl phthalate (DEHP), and found that its metabolite, mono-2-ethylhexyl phthalate (MEHP), had significant adverse effects on myeloid hematopoiesis at environmentally relevant concentrations. An analysis of the underlying mechanism revealed that MEHP promotes increases in reactive oxygen species (ROS) by reducing the activity of superoxide dismutase in all lineages, possibly via its actions at the aryl hydrocarbon receptor. This leads to a metabolic shift away from glycolysis toward the pentose phosphate pathway and ultimately results in the death of hematopoietic cells that rely on glycolysis for energy production. By contrast, cells that utilize fatty acid oxidation for energy production are not susceptible to this outcome due to their capacity to uncouple ATP production. These responses were also detected in non-hematopoietic cells exposed to alternate inducers of ROS.

## 1. Introduction

Hematopoiesis is a fundamental process in the bone marrow that is responsible for the continuous production of all blood cells throughout human life [1]. Thus, hematopoiesis is particularly vulnerable to environmental insults, most notably those resulting from exposure to endocrine-disrupting chemicals (EDCs), which are ubiquitous in the environment [2]. Thus, research focused on the impact of these compounds on human hematopoietic stem and progenitor cell (HSPC) differentiation is highly relevant and of great importance [3,4].

Di-2-ethylhexyl phthalate (DEHP) belongs to the class of EDCs that are commonly used as plasticizers in the manufacture of commercial polymer products. Upon contact, DEHP leaches into liquids due to its comparatively weak, non-covalent binding interactions with the manufactured material, raising significant concerns about its effects on human health [5,6]. For example, DEHP concentrations as high as 50 to 70 mg/L were found in stored blood bags [7]. Neonates in intensive care units were identified as among those who were highly exposed to this EDC, with serum DEHP levels reaching 123.1 µg/mL after completion of a transfusion [7,8]. However, DEHP is rapidly metabolized in the human body with a half-life of 6–12 h [9,10,11]. Moreover, several studies suggest that DEHP exerts much of its toxicity via the actions of its first metabolite, mono-2-ethylhexyl phthalate (MEHP). Apart from acute high exposure settings, lower levels of DEHP (from 0.1 to 2.7 µg/mL) have been detected in human blood samples [12,13,14,15,16]. This was frequently accompanied by the detection of MEHP at concentrations from 0.13 to 0.68 µg/mL, most likely reflecting baseline exposure levels [12,13,15,16].

The extent of DEHP-mediated toxicity, as well as general effects associated with exposure, differs depending on the target cell type under study [5,7,17,18,19,20,21,22]. With respect to the mechanism, most of these effects appear to be mediated via DEHP-dependent activation of the peroxisome proliferator-activated receptor-gamma (PPARγ) or the aryl hydrocarbon receptor (AhR) followed by the generation of reactive oxygen species (ROS) [5,7,21,22,23,24,25,26]. From an epidemiological perspective, exposure to phthalates is associated with the development of allergic symptoms in children [27]. As DEHP-mediated modulation of dendritic cell differentiation from peripheral blood mononuclear cells has been observed, it is reasonable to consider the possibility that DEHP may have a profound impact on human hematopoiesis [25,28,29]. A recent study focused on the effects of DEHP on hematopoietic cell development and reported decreases in the abundance of colony-forming units (CFUs) and changes in colony composition [30]. These observations are of particular importance since they were detected after short-term exposure to DEHP and thus largely reflect the conditions found in an intensive care unit [30]. However, there is very little information available that provides insight into the biochemical mechanisms underlying these derangements. As the search for alternative plasticizers continues, it will be critical to have an adequate understanding of the pathophysiologic responses to DEHP in order to have the means to evaluate the impact of any prospective alternative compounds.

We recently established and characterized a model of myeloid HSPC differentiation capable of undergoing differentiation into erythrocyte, dendritic cell, and neutrophil lineages. Metabolic activities differed markedly between these lineages; thus, this model will be ideal for use in experiments designed to characterize the effects of individual compounds on specific signaling and metabolic pathways and will also facilitate the identification of common mechanisms [31]. The use of this model could also lead to the identification of potentially unique protective mechanisms; this would not be possible in experiments featuring single cell lines. We here examined the impact of DEHP on HSPC differentiation into cells of the erythrocyte, dendritic cell, and neutrophil cell lineages. We characterized these responses and identified the underlying mechanisms. As the impact of DEHP was fundamentally different in neutrophils, we examined the impact of DEHP on the energy metabolism. We used this information to examine the impact of DEHP administration in various cell lines and responses to other substance classes. Taken together, we provide strong evidence that the contribution of DEHP in the development of allergic disease already occurs at the hematopoietic level subject to mediation by other environmentally relevant substances through the generation of ROS.

## 2. Materials and Methods

A detailed list of all reagents and materials used in this study, including manufacturers and identifying data, can be found in Supplementary Table S1.

### 2.1. Human CD34^+^ Cell Culture

Human cord blood CD34^+^ cells were expanded in Stempro-34 serum-free medium (SFM) containing 2 mM L-glutamine, 100 ng/mL stem cell factor (SCF), 100 ng/mL Flt-3 ligand (Flt-3L), 50 ng/mL interleukin (IL)-6, 40 ng/mL thrombopoietin (TPO), 1 µM StemRegenin (SR)-1, 0.1 µM BML-210, and 0.2 mM valproic acid as previously described [31]. Erythroid differentiation was induced in Stempro-34 SFM containing 2 mM L-glutamine, 100 ng/mL SCF, 100 ng/mL Flt-3L, 50 ng/mL erythropoietin (EPO), and 20 ng/mL IL-3 for up to 6 days as previously described [31]. Dendritic cell differentiation was induced in Stempro-34 SFM containing 2 mM L-glutamine, 10 ng/mL SCF, 10 ng/mL TPO, and 10 ng/mL IL-3 for up to 11 days as previously described [31]. Neutrophil differentiation was induced in Iscove’s Modified Dulbecco’s Medium (IMDM) containing 10% fetal bovine serum, 50 ng/mL SCF, and 50 ng/mL IL-15 for up to 10 days as previously described [31]. DEHP, MEHP, or 2-ethylhexanol (2-EH), all dissolved in ethanol, pure ethanol (vehicle control), or agonists/antagonists as indicated, was added to the differentiating cultures and maintained at constant concentrations during the experiments. The added volume did not exceed 0.1% (*v*/*v*). Cell viability was assessed on a routine basis using the trypan blue exclusion method.

### 2.2. HUVEC and HepG2/C3A Cell Culture

Human umbilical vein endothelial cells (HUVECs) were cultured in endothelial cell growth medium at 37 °C in a humidified atmosphere (5% CO_2_/95% air). HepG2/C3A cells were cultured in Dulbecco’s Modified Eagle Medium (DMEM; without phenol red) supplemented with 10% fetal bovine serum, 100 U/mL penicillin, and 100 mg/mL streptomycin at 37 °C in a humidified atmosphere (5% CO_2_/95% air). Cells were seeded into 96-well plates at a density of 4 × 10^4^ cells/well and permitted to attach overnight before initiating the experiments.

### 2.3. Flow Cytometry

All flow cytometry data were acquired on a CyFlow Cube 8 flow cytometer (Sysmex) and analyzed using FCS Express software (De Novo Software). Cells were washed twice with Dulbecco’s phosphate-buffered saline (dPBS) and blocked with 10% human male AB serum for 15 min at 4 °C as previously described [32]. The cells were then double-stained with anti-human antibodies as indicated using concentrations and incubation times provided by the manufacturers’ instructions. After incubation, cells were washed twice with dPBS and analyzed immediately by flow cytometry.

### 2.4. Caspase 3/7 Activation

Erythrocytes were differentiated in the presence of varying concentrations of DEHP for 2 days. Dendritic cells were differentiated for 3 days. Then, 1 µM of Staurosporine was added for 18 h. Before performing the assay, cell suspensions were diluted to 1 × 10^5^/mL with pre-warmed media. Caspase 3/7 activation was measured using the Apo-ONE Homogenous Caspase-3/7 Assay (Promega, Walldorf, Germany) following the manufacturer’s protocol. Cells were incubated with the reagent for 1 h at room temperature.

### 2.5. ATP Quantification

Absolute levels of ATP per 10^5^ cells were determined using the ATP Detection Assay Kit—Luminescence (Cayman Chemical, Ann Arbor, MI, USA). Briefly, hematopoietic cell lineages were differentiated in the presence of DEHP for 2 days (for erythrocytes) or 3 days (for dendritic cells and neutrophils). Cells were then washed twice with PBS and resuspended at 1 × 10^5^ cells in a 167 µL ATP detection sample buffer. Cell lysis and ATP quantitation were performed following the manufacturer’s protocol.

### 2.6. NADPH Quantification

NADPH levels per 5 × 10^5^ cells were determined using the Colorimetric NADPH Assay Kit (Abcam, Cambridge, UK). Briefly, hematopoietic cell lineages were differentiated in the presence of DEHP for 2 days (for erythrocytes) or 3 days (for dendritic cells or neutrophils). Cells were then washed twice with PBS at 4 °C and resuspended at 1 × 10^6^ cells per 100 µL of the Lysis buffer. Cell lysis and NADPH quantitation was performed following the manufacturer’s protocol. Lysates were incubated with the reagent for 1 h at room temperature.

### 2.7. H_2_DCFDC-Assay for Detection of ROS

Relative levels of cellular ROS were determined by using 2′-7′-dichlorodihydrofluorescein-diacetate (H_2_DCFDA) as previously described [5]. Briefly, dendritic cells and neutrophils were differentiated for 3 days in the absence or presence of various concentrations of DEHP, MEHP, or 2-EH as indicated. Erythrocytes were differentiated for 2 days prior to treatment with various concentrations of DEHP, MEHP, or 2-EH for 4 h as indicated. HUVECs and HepG2/C3A cells were treated for 4 h with DEHP or tert-butyl hydroperoxide (TBHP). Etomoxir (ETO), N-acetylcysteine (NAC), or butylated hydroxyanisole (BHA) was added 2 h prior to treatment with DEHP or TBHP. Following DEHP or TBHP treatment, the ROS-detecting agent, H_2_DCFDA, was added to each well to a final concentration of 20 µM. Cultures were then incubated at 37 °C for 30 min. After incubation, cells were washed twice with PBS and resuspended at 1 × 10^5^ cells/100 µL. The production of oxidized dye, detected as DCF fluorescence, was measured using a plate reader at Ex/Em wavelengths of 485 nm/535 nm, respectively.

### 2.8. Isolation of Total RNA and qPCR

The isolation of total RNA was performed using the NucleoSpin RNA XS Kit (Macherey-Nagel GmbH & Co. KG, Düren, Germany). First-strand complementary DNA (cDNA) synthesis was performed using 650 ng of total RNA as previously described [31]. Real-time PCR was performed in a LightCycler 480 (Roche Diagnostics, Mannheim, Germany), using the LightCycler 480 SYBR Green I Master Kit (Roche Diagnostics, Mannheim, Germany) according to the manufacturer’s instructions. Raw data (Ct-values) were determined using LightCycler 480 SW software (version 1.5; Roche Diagnostics). Ribosomal Protein Lateral Stalk Subunit P0 (RPLP0) was chosen as the reference gene for the normalization of gene expression. Relative gene expression was calculated using the 2^−ΔΔCt^ method [33]. Primer sequences are listed in Supplementary Table S1.

### 2.9. LC-MS-Based Metabolomics

Cell pellets were collected at the times indicated and extracted as previously described [31]. Metabolomics analyses were carried out with the AbsoluteIDQ p180 Kit (Biocrates Life Science AG, Innsbruck, Austria) according to the manufacturer’s instructions. Sphingolipids, including sphingosine, sphingosine-1-phosphate, as well as C16-, C18-, C20-, C24-ceramide and their corresponding dihydro-species, as well as lysosphingomyelin d18:1 and C16-sphingomyelin, were quantified using liquid chromatography (LC)-mass spectrometry (MS). Briefly, 10 µL of an internal standard solution, consisting of 437.9 nM sphingosine d17:1, 684 nM sphingosine-1-phosphate d17:1, 477.1 nM C15 ceramide, and 1.25 µM C24 ceramide d17:1, were applied to punches of 0.75 mm blotting paper (7 mm diameter) immobilized in a 96-well filter plate placed over a 96-deep-well plate and dried under nitrogen flow (40 psi) for 20 min. Next, 20 µL of calibrators or cell extracts were applied to each blotting paper punch and dried under nitrogen flow for 40 min. Metabolites of interest were resolved in 300 µL of methanol/methyl tert-butyl ether (MTBE) at an 80:20 ratio for 30 min on a horizontal shaker at 450 rpm and 20 °C followed by a transfer to the deep well plate via centrifugation at a 500 relative centrifugal force (rcf) for 5 min at 4 °C. The extracts were evaluated by LC-MS with the autosampler set at a temperature of 10 °C while measurements were collected.

Chromatographic separations were performed on a NexeraXR HPLC (Shimadzu, Duisburg, Germany) using a C8 Ultra-Inert HPLC column (50 mm L × 2.1 mm internal diameter (i.d.), 3 µm particle size; ACE) in combination with a C8 Ultra-Inert HPLC guard cartridge (10 mm L × 2.1 mm i.d., 3 µm particle size; ACE) preheated to 50 °C. The mobile phase included eluent A (water with 0.4% formic acid) and eluent B (2-propanol with 0.4% formic acid) that were applied to the column as follows. Eluent A/B at an 80:20 ratio was applied from t = 0 to 0.5 min followed by a linear gradient of 80:20 to 0:100 A/B from t = 0.5 to 4.5 min. This was followed by eluent A/B at 0:100 from t = 4.5 to 6 min, and finally A/B at 80:20 from t = 6 to 8 min. The injection volume was 20 µL, and the flow rate was 1 mL/min throughout. A typical chromatogram is shown in Supplementary Figure S1, and the corresponding assay parameters for each analyte are presented in Supplementary Table S2. The 4000 QTRAP mass spectrometer (AB Sciex, Framingham, MA, USA) was operated in the positive ion mode with an electrospray voltage of 5000 V at 450 °C, curtain gas at 25 psi, collision gas at 6 psi, nebulizing gas at 25 psi, and auxiliary gas at 25 psi. All quadrupoles were working at unit resolution.

Typical precursor-to-product ion transitions (as per [34]), with the corresponding parameters and retention times as shown in Supplementary Table S2, were used as quantifiers for the scheduled multiple reaction monitoring (MRM), and the retention time window was set at 60 s. Quantitation was performed using the MultiQuant V3.0.3 Software (ABI Sciex, Framingham, MA, USA) using a 7-point calibration for each analyte within the concentration ranges indicated in Supplementary Table S3. Ratios of analyte peak area and internal standard peak area were plotted against calibrator concentrations, and calibration curves were calculated by least-squares quadratic regression with 1/x weighting.

### 2.10. Western Blot Analysis

Cells were seeded in 6-well plates for Western blot analysis. Lineages were differentiated in the presence of indicated concentrations of DEHP for 2 days (for erythrocytes) and 3 days (for dendritic cells and neutrophils). Cells were washed twice with ice-cold PBS and lysed in ice-cold radioimmunoprecipitation assay (RIPA) buffer containing protease and phosphatase inhibitors (0.5 mg/mL Pefabloc and 1× PhosSTOP, respectively) at 50 µL per 1 × 10^6^ cells at 4 °C for 30 min. Following lysis, lysates were cleared by centrifugation at 15,000 rcf for 20 min at 4 °C. The protein content was determined using the bicinchoninic acid (BCA) Protein Assay Kit (Thermo Fisher Scientific, Schwerte, Germany). Twenty µg of total protein were subjected to electrophoresis on a sodium dodecyl sulfate-polyacrylamide gel (SDS-PAGE; 4% stacking and 8% separation gels) and transferred to nitrocellulose membranes using a Bio-Rad Mini Trans-Blot apparatus. Membranes were blocked with 5% (*w*/*v*) bovine serum albumin (BSA) in Tris-buffered saline with Tween (TBS-T) for 45 min. Membranes were then incubated overnight at 4 °C with mouse anti-AhR, anti-Cyp1a1, or anti-Cyp1b1 antibodies at the dilutions recommended by the manufacturer. After incubation, the membranes were washed three times with TBS-T and then incubated for 1 h with horseradish peroxidase (HRP)-conjugated secondary anti-mouse antibody (1:1250) at room temperature. Signals were detected using the SuperSignal West Pico PLUS chemiluminescent substrate (Thermo Fisher Scientific) and visualized with ChemStudio PLUS (Analytik Jena GmbH, Jena, Germany). Band intensities were quantified by densitometry using ImageJ 1.53a software [35]. After the detection of the signal from a first primary antibody, membranes were stripped using ROTI Free Stripping-Buffer 2.2 plus (Carl Roth GmbH & Co. KG, Karlsruhe, Germany) for 20 min at room temperature, followed by washing with TBS-T and blocking prior to incubation with another primary antibody.

### 2.11. Measurement of Superoxide Dismutase (SOD) Activity

Relative SOD activity was determined using the colorimetric Superoxide Dismutase Activity Assay Kit (Abcam, Cambridge, UK) following the manufacturer’s instructions. Briefly, hematopoietic cell lineages were differentiated in the presence of DEHP or MEHP for 2 days (for erythrocytes) or 3 days (for dendritic cells or neutrophils). Cells were washed twice with PBS at 4 °C and resuspended at 1 × 10^6^ cells per 50 µL lysis buffer. SOD activity was assessed in a 10 µg sample of total protein extract as previously described [36].

### 2.12. GC-MS-Based Quantification of DEHP and MEHP in Culture Medium

The concentrations of DEHP and its primary metabolite, MEHP, in the culture medium were determined by gas chromatography-mass spectrometry (GC-MS) as previously described [37]. Briefly, 5 µL of an internal standard solution consisting of 7.185 mM MEHP-d4 and 5.068 mM DEHP-d4 were added to 1 mL of the culture media or standard solutions. This was followed by the addition of 200 µL of ammonium acetate buffer (pH 6.5) and 10 µL of β-glucuronidase. The mixture was incubated at 37 °C for 90 min on a horizontal shaker. Samples were then acidified by the addition of 50 µL 37% hydrochloric acid and immediately extracted with 8 mL MTBE for 20 min on a tumble shaker. After centrifugation for 5 min at 3000 rcf, the upper phase was transferred to fresh vials and evaporated to dryness in a vacuum centrifuge. The dried residue was resuspended in 100 µL acetonitrile and 10 µL of pentafluorobenzyl bromide, and 10 µL of triethylamine were added. The mixture was then heated to 75 °C for 20 min; 2 mL of sodium acetate buffer (pH 5.0) and 1 mL of hexane were added after cooling. The mixture was shaken for 2 min, and 750 µL of the upper hexane phase were transferred to a 2 mL autosampler vial. The GC-MS analysis was performed on a Clarus 600T instrument (Perkin Elmer, Rodgau, Germany) using a Rxi-5Sil MS GC column (30 mL × 0.25 mm i.d., 0.25 µL film thickness) and hydrogen (2 mL/min) as the carrier gas. The injector temperature was 250 °C, and the sample volume was 2 µL. The column was initially heated to 70 °C for 3 min, then increased at a rate of 13 °C/min to 280 °C; the temperature was held at 280 °C for 15.83 min. DEHP, MEHP, and deuterated internal standards were detected in the SIM mode using 149 m/z or 153 m/z fragments as quantifiers as previously described [38,39]. All materials used for this analysis were tested for the absence of DEHP and MEHP prior to use.

### 2.13. Quantification and Statistical Analysis

All data are represented as indicated in the legends corresponding to each figure. The statistical analysis of metabolomics data was performed using an empirical Bayes approach using statistical software in R [40] and the Bioconductor package limma [41]. An absolute log_2_ fold change (logFC) above 0.5, with a corresponding false discovery rate (FDR)-adjusted *p*-value below 0.05 (as per the Benjamini–Hochberg procedure), was considered relevant. A one-way ANOVA followed by Tukey’s multiple comparison procedure was performed as indicated; *p*-values below 0.05 were considered to be statistically significant. Cell growth is presented as a fold expansion, which was calculated as the final cell number divided by the initial cell number for all experiments. Kyoto Encyclopedia of Genes and Genomes (KEGG) [42] pathways were used as a template for metabolic pathway visualization as per PathViso [43] software. Schemes were created using BioRender.

## 3. Results

### 3.1. DEHP Induces Erythrocyte and Dendritic Cell Apoptosis While Enhancing Neutrophil Maturation

**Figure 1:**
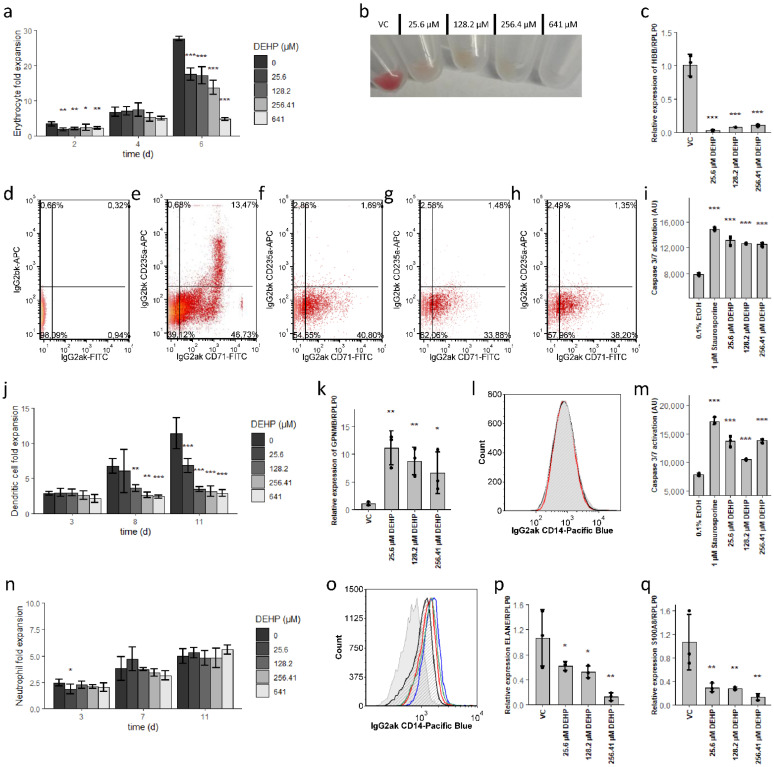
Differential disruption of myeloid lineage differentiation by DEHP. (**a**,**j**,**n**) Expansion of erythrocyte (**a**), dendritic cell (**i**), and neutrophil (**n**) cultures in the presence of increasing concentrations of DEHP. (**b**) Cell pellet representing erythroid populations after 6 days of treatment with increasing concentrations of DEHP. (**c**) Impact of increasing concentrations of DEHP on the relative expression of HBB in differentiating erythrocytes after 6 days. (**d**–**h**) Expression of CD71 and CD235a in erythroid populations after 6 days of treatment with varying concentrations DEHP. From left to right: isotype control (**d**), vehicle control (**e**), 25.6 µM (**f**), 128.2 µM (**g**), and 256.41 µM DEHP (**h**). (**i**,**m**) Caspase activity in differentiated erythroid populations after 2 days (**i**) and in dendritic cell populations after 3 days (**m**) of treatment with varying concentrations of DEHP. Cells treated with staurosporine were included as positive controls. (**k**) Impact of DEHP on the relative expression of GPNMB in dendritic cell cultures. (**l**) Expression of CD14 on dendritic cells treated with 256.41 µM DEHP (red) compared to vehicle control (black); isotype control is as shown (grey-filled). (**o**) Expression of CD14 on neutrophils treated with 25.6 µM (red), 128.2 µM (green), and 256.41 µM DEHP (blue) compared to vehicle control (black); isotype control is as shown (grey). (**p**,**q**) Impact of DEHP on the relative expression of ELANE (**p**) and S100A8 (**q**) in neutrophils. Data are presented as mean ± standard error of the mean (SEM); individual replicates are visualized as black dots in bar charts in case of *n* = 3. Significance was determined by one-way ANOVA (* *p* < 0.05; ** *p* < 0.01; *** *p* < 0.001).

Large quantities of all blood cells are formed from just a few progenitor cells. Thus, a large expansion of cell numbers accompanies differentiation into the individual hematopoietic lineages. Therefore, as a first step, we examined the impact of increasing concentrations of DEHP (25.6 µM (10 µg/mL), 128.2 µM (50 µg/mL), 256.41 µM (100 µg/mL), and 641 µM (250 µg/mL)) on the rate of cell proliferation of the various lineages at different time points using our established hematopoiesis model [31]. The addition of DEHP significantly reduced the rates of proliferation of differentiating erythroid and dendritic cells, with effects that were more pronounced at higher concentrations (Figure 1a,j). Interestingly, DEHP had no impact on the proliferation of differentiating neutrophils (Figure 1n). Surface markers of early (CD71) and late (CD235a) stages of erythroid differentiation and expression levels of hemoglobin subunit beta (HBB) were also drastically reduced in response to DEHP.

641 µM (250 µg/mL)) on the rate of cell proliferation of the various lineages at dif-ferent time points using our established hematopoiesis model [31]. The addition of DEHP significantly reduced the rates of proliferation of differentiating erythroid and dendritic cells, with effects that were more pronounced at higher concentrations (Figure 1a,j). Interestingly, DEHP had no impact on the proliferation of differentiating neutrophils (Figure 1n). Surface markers of early (CD71) and late (CD235a) stages of erythroid differentiation and expression levels of hemoglobin subunit beta (HBB) were also drastically reduced in response to DEHP.

These DEHP-mediated reductions were detected prominently on day six in the erythrocyte culture (Figure 1c–h) and were accompanied by the absence of reddish staining of the cell pellets (Figure 1b) [44]. By contrast, treatment with DEHP resulted in an increased expression of *GPNMB*, a cell surface glycoprotein characteristic of antigen-presenting cells (APCs; Figure 1k) [45]. As *GPNMB* is also expressed by monocytes and macrophages, we also evaluated the expression of the monocyte antigen, CD14, which remained unaffected in response to treatment with 256.41 µM DEHP (Figure 1l). Thus, an increased expression of *GPNMB* does not appear to be reflecting a shift towards monocytic differentiation [46]. We also detected DEHP-dependent increases in caspase 3/7 activity (Figure 1i,m) which suggested that the reduced proliferation detected in erythrocyte and dendritic cell cultures was most likely to result from increasing rates of apoptosis. By contrast, DEHP had no impact on caspase 3/7 activity in differentiating neutrophil cultures (results not shown), a finding that parallels their ongoing proliferation. However, the addition of DEHP resulted in the reduced expression of the functional neutrophil lineage related genes *ELANE* and *S100A8* (Figure 1p,q). Of note, the expression of the genes encoding *ELANE* and *S100A8* is maturation-stage dependent. *ELANE* expression decreases while *S100A8* expression increases at the metamyelocyte stage; the expression of *S100A8* then decreases as the polymorphonuclear neutrophils mature [47]. Thus, the DEHP-mediated expression pattern represents either severe inhibition or increased rates of neutrophil maturation. As the proportion of CD14^low^ cells (i.e., more mature neutrophils) increases in response to DEHP, the latter mechanism (i.e., increased rates of neutrophil maturation) is most likely to be the case (Figure 1o) [47,48].

In summary, our data demonstrate that DEHP disrupts erythrocyte and dendritic cell maturation via the selective induction of apoptosis. Interestingly, neutrophil maturation appears to be enhanced in response to treatment with DEHP.

### 3.2. DEHP Alters the Lipidome Composition and Reduces the Rates of Glycolysis, Glutaminolysis, and Polyamine Synthesis in Erythrocytes and Dendritic Cells

As DEHP had unique effects on the growth and differentiation of individual myeloid cell lineages, we sought to identify the mechanisms underlying these opposing actions. As the metabolism plays a central role in promoting lineage commitment as well as the general cellular response to exogenous stimuli, we performed a quantitative analysis of 200 cellular metabolites in differentiating cells, including amino acids, biogenic amines, acylcarnitines, glycerophospholipids (GPLs), sphingolipids, and total hexose sugars [31,49,50]. Several of these metabolites were selected based on known differentiation-dependent changes that were identified as part of the characterization of the original model; this strategy enabled us to predict the relative activity of their respective metabolic pathways. However, since DEHP promoted increased rates of apoptosis in both erythrocyte and dendritic cell cultures, we also included a focus on the ceramide metabolism [51,52]. The identification of the mechanisms underlying potentially elevated ceramide concentrations (e.g., de novo synthesis in the endoplasmic reticulum vs. hydrolysis of sphingomyelin in lysosomes or the mitochondria) might provide us with immediate insight into the mechanisms underlying apoptosis in these culture systems [53,54,55].

**Figure 2:**
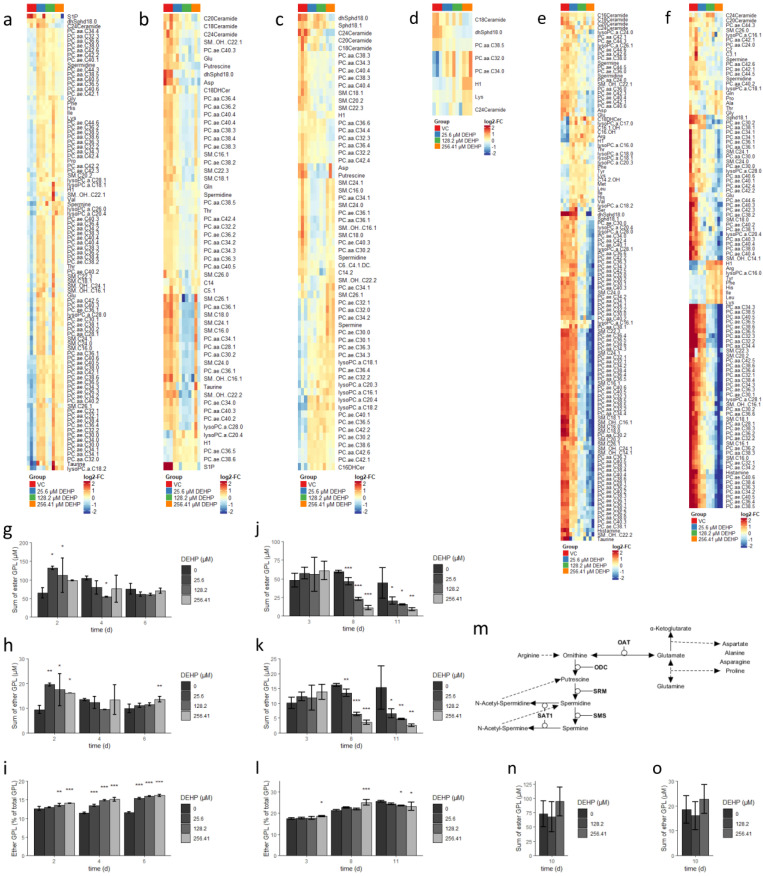
DEHP alters the lipidome and suppresses glycolysis, glutaminolysis, and polyamine synthesis in erythrocytes and dendritic cells. (**a**–**c**) Erythrocyte metabolites altered (FDR-significant) after 2 days (**a**), 4 days (**b**), and 6 days (**c**) in culture with increasing concentrations of DEHP (**d**–**f**) Dendritic cell metabolites (FDR-significant) after 3 days (**d**), 8 days (**e**), and 11 days (**f**) in culture with increasing concentrations of DEHP. (**g**) Total ester glycerophospholipids (GPL) concentration in erythroid cultures treated with increasing concentrations of DEHP. (**h**) Total ether GPL concentration in erythroid cultures treated with increasing concentrations of DEHP. (**i**) Percent ether GPL content in erythroid cultures treated with increasing concentrations of DEHP. (**j**) Total ester GPL concentration in dendritic cell cultures treated with increasing concentrations of DEHP. (**k**) Total ether GPL concentration in dendritic cell cultures treated with increasing concentrations of DEHP. (**l**) Percent ether GPL content in dendritic cell cultures treated with increasing concentrations of DEHP. (**m**) Model depicting the interconnections between altered metabolites associated with glutaminolysis and polyamine synthesis. (**n**) Total ester GPL content in neutrophil cultures treated with increasing concentrations of DEHP. (**o**) Total ether GPL content in neutrophil cultures treated with increasing concentrations of DEHP. Data are presented as mean ± SEM. Statistical significance was determined by one-way ANOVA (* *p* < 0.05; ** *p* < 0.01; *** *p* < 0.001).

As shown in Figure 2a–c, we identified significant DEHP-dependent alterations in metabolite concentrations in the differentiating erythroid cultures, including a total of 92 metabolites at day 2, 51 metabolites at day 4, and 55 metabolites at day 6 (see also Supplementary Dataset S1). Among these findings, we identified significant DEHP-dependent elevations in the levels of acyl-acyl phosphatidylcholines (PC; “PC.aa.” species) and sphingolipids (SL; “SM”, “Ceramide”, and “Sph” species) on day 2; these elevated levels were reduced on days 4 and 6. Additionally, several ether phosphatidylcholines (EPCs; “PC.ae.” species) increased at days 2 and 6 with reductions observed on day 4.

Metabolites at day 2, 51 metabolites at day 4, and 55 metabolites at day 6 (see also Supplementary Dataset S1). Among these findings, we identified significant DEHP-dependent elevations in the levels of acyl-acyl phosphatidylcholines (PC; “PC.aa.” species) and sphingolipids (SL; “SM”, “Ceramide”, and “Sph” species) on day 2; these elevated levels were reduced on days 4 and 6. Additionally, several ether phosphatidylcholines (EPCs; “PC.ae.” species) increased at days 2 and 6 with reduc-tions observed on day 4.

We also detected elevated concentrations of several lysophosphatidylcholines (lysoPC; “lyso.PC.a.” species) at all time points. As we previously identified high rates of fatty acid (FA) synthesis in combination with low rates of FA oxidation (FAO) as a mechanism underlying increased levels of GPLs and SLs detected in erythrocytes, it is reasonable to assume that these outcomes are the result of the initial stimulation of FA and GPL synthesis by DEHP [31]. This hypothesis is further supported by observed DEHP-dependent increases in total ester and ether GPLs detected at day 2 in the culture (Figure 2g,h).

Elevated levels of several lysoPCs together with reduced levels of SLs (from day four onward) suggest their enhanced degradation in later stages of differentiation. As phospholipases that target ester GPLs are often incapable of hydrolyzing the corresponding ether GPL species, EPCs remain unaffected by this process. This is also reflected by their increased fractional (i.e., percentage) content detected at later time points (Figure 2i) [56]. However, we found that DEHP had no impact on acylcarnitine levels. This finding suggests that total cellular acyl-CoA and thus free FA levels were not altered in response to DEHP; thus, it is likely that the cells respond with reduced rates of FA synthesis as well.

We identified DEHP-dependent changes in levels of only five metabolites at day 3 in cultures of differentiating dendritic cells; by contrast, levels of 115 and 99 metabolites were altered at day 8 and 11, respectively (Figure 2d–f, also Supplementary Dataset S1). Interestingly, we detected DEHP-concentration-dependent reductions in the levels of most of the PCs, EPCs, and SLs from day 8 onward. This finding was also reflected by the total measured levels of ester and ether GPLs, which were both remarkably reduced in dendritic cell cultures treated with DEHP (Figure 2j,k). However, DEHP had no substantial impact on the fractional content of ether lipids (Figure 2l). Taken together, our data indicate that exposure to DEHP resulted in the reduced synthesis of both GPLs and SLs in dendritic cell cultures, as the levels of most of the lysoPCs were reduced under these conditions.

Our findings also revealed DEHP-dependent increases in the levels of hexose sugars in both erythrocyte and dendritic cell cultures at all time points tested. These results suggested that exposure to DEHP results in the reduction in the glucose catabolism. Furthermore, altered levels of glutamine, glutamate, proline, asparagine, and aspartate, as well as arginine, putrescine, spermidine, and spermine, indicate a DEHP-mediated disruption of glutaminolysis and polyamine synthesis in both lineages (Figure 2a–f). While we identified a DEHP-dependent shift from glutamate conversion to α-ketoglutarate toward the anabolism of other products, including polyamines at day 2 in erythrocyte cultures, the reduced glutamine uptake appears to be responsible for the reduced levels of related metabolites observed at later time points (see the model in Figure 2m). By contrast, we detected a DEHP-dependent diminished conversion of arginine to ornithine in dendritic cell cultures, together with the enhanced conversion of glutamate to proline. Taken together, these findings suggest that exposure to DEHP results in reduced rates of glycolysis, glutaminolysis, and polyamine synthesis in both erythroid and dendritic cell lineages.

Interestingly, ceramide levels were reduced rather than increased in both the erythroid and dendritic cell lineages. This observation suggests that apoptosis was not dependent on ceramide concentrations. As an increase in ceramide levels has been associated with numerous extrinsic stimuli (i.e., receptor-mediated) as well as with intrinsic mitochondrial apoptosis, we can conclude that DEHP does not activate either of these pathways [56]. However, previous studies established that apoptosis resulting from NF-κB activation or increased endoplasmic reticulum stress is not dependent on elevated ceramide levels [57,58,59]. Thus, we hypothesize that DEHP promotes apoptosis via one of these mechanisms.

By contrast, the administration of DEHP at 128.2 µM (50 µg/mL) or 256.41 µM (100 µg/mL) for 10 days resulted in no metabolite alterations in differentiating neutrophil cultures; total ester GPLs and total ether GPLs also remained unchanged (Figure 2n,o). Given that (1) the administration of DEHP resulted in major metabolic alterations of the lipidomes of differentiating erythroid and dendritic cells, and (2) of this cohort, neutrophils are the only lineage capable of active FAO; there may be a relationship between the resistance to the effects of DEHP and FAO activity [31,50,60].

### 3.3. MEHP, the First Metabolite of DEHP, Was Detected at Environmentally Relevant Concentrations and Induced the Accumulation of ROS via the Reduction of SOD Activity

The results of the metabolome analysis revealed that DEHP had a significant impact on the lipid metabolism of both erythroid and dendritic cell lineages. Changes in the lipidome can be the result of either altered catabolic or anabolic responses. The activation of an anabolic response has been associated with increased concentrations of free fatty acids and thus acylcarnitines [61]. However, levels of most of the acylcarnitine species measured were not altered during the time course of these experiments. These results suggested that acylcarnitines were either degraded in response to DEHP-mediated activation of FAO or that rates of the synthesis of the corresponding CoA (and thus carnitine) species were reduced. Because the regulation of FAO was previously implicated in the HSPC fate decision, and the DEHP metabolite, MEHP, promoted an increased expression of genes associated with this process, we evaluated the possibility that DEHP disturbs the lipid metabolism via its capacity to upregulate FAO [31,50,62]. We also noted the documented interaction of DEHP and MEHP with the receptors peroxisome proliferator-activated receptor gamma (PPARγ) and alpha (PPARα) which are both involved in the differential regulation of the lipid metabolism [5,23,24,63,64,65,66]. Therefore, we also determined whether the actions of DEHP were mediated via enhanced FAO and the activation of these receptors via studies that feature the antagonists etomoxir, GW-9662, and GW-6471 that target carnitine palmitoyltransferase 1A (CPT1a), PPARγ, and PPARα, respectively. As shown in Supplemental Figure S2, none of these antagonists restored proliferation rates or gene expression patterns in differentiating erythroid or dendritic cells despite positive controls that confirmed their inhibitory effects on PPARα/γ activation in both lineages (Supplemental Figure S2). Therefore, we concluded that the observed lineage selective responses to DEHP were not mediated by enhanced FAO or by the activation of PPARs.

**Figure 3:**
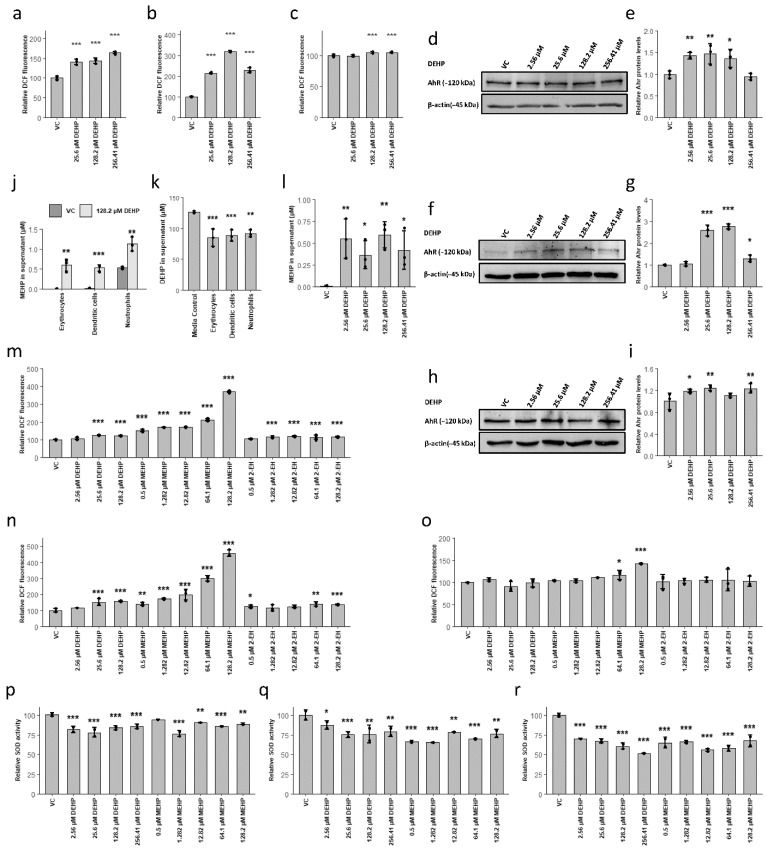
The DEHP metabolite, MEHP, reduces SOD activity and increases ROS at environmentally relevant concentrations. (**a**–**c**) Relative fluorescence (DCF) after DEHP treatment of erythrocytes for 4 h (**a**), dendritic cells for 3 d (**b**), and neutrophils for 3 d (**c**). (**d**–**i**) Relative expression of aryl hydrocarbon receptor (AhR) protein in erythrocytes (**d**,**e**), dendritic cells (**f**,**g**), and neutrophils (**h**,**i**) in response to increasing concentrations of DEHP. (**j**–**l**) Concentrations of MEHP (**j**) and DEHP (**k**) detected in cell culture supernatants after treatment with 128.2 µM DEHP and other concentrations for 48 h (**l**). (**m**–**o**) Relative fluorescence (DCF) after MEHP or 2-EH treatment of erythrocytes for 4 h (**m**), dendritic cells for 3 d (**n**), and neutrophils for 3 d (**o**). (**p**–**r**) Relative SOD activity after DEHP or MEHP treatment of erythrocytes for 2 d (**p**), dendritic cells for 3 d (**q**), and neutrophils for 3 d (**r**). Data are presented as mean ± standard error of the mean (SEM); individual replicates are visualized as black dots in bar charts in case of *n* = 3. Significance was determined by one-way ANOVA (* *p* < 0.05; ** *p* < 0.01; *** *p* < 0.001).

Given these findings, we focused on ROS generation, which was another prominent response to DEHP. While profoundly increased levels of DEHP-induced oxidative stress were detected in both erythrocyte and dendritic cell cultures, neutrophils displayed only a slight increase (Figure 3a–c). Mechanistically, enhanced ROS formation in response to DEHP is linked to the activation of the aryl hydrocarbon receptor (AhR), leading to increased activity of both Cyp1a1 and Cyp1b1 [7,26,29]. In support of these findings, we identified DEHP-mediated increases in AhR protein levels when administered at concentrations as low as 2.56 µM (1 µg/mL). Surprisingly, this response was also observed in neutrophils (Figure 2d–i). However, the expression and enzymatic activity of AhR downstream mediators Cyp1a1 and Cyp1b1 could not be confirmed by Western blot or by an ethoxyresorufin O-deethylase assay in any of the cell lineages (results not shown). Importantly, a recent study documented that MEHP can inhibit Cyp1a1 expression via an antagonistic effect on AhR [67]. Therefore, we hypothesized that the observed effects may be the result of exposure to the MEHP metabolite of DEHP and not by the parent compound. To this end, the conversion of DEHP to MEHP was confirmed in all lineages. MEHP was detected at 0.566 ± 0.129 µM (0.157 ± 0.036 µg/mL) in the supernatants of erythroid, dendritic cell, and neutrophil cultures, which are concentrations that correspond to those previously reported in other in vitro models (Figure 3j) [5,7]. Interestingly, the rate of the DEHP metabolism to MEHP was not an accurate reflection of conditions characterized in vivo. Specifically, these results indicate that only ~30% of the added DEHP was degraded after >48 h of incubation. Furthermore, the amount of MEHP detected was not dependent on the concentration of the parent compound (Figure 3j,k). Thus, we assume that the metabolism of DEHP proceeded somewhat more slowly in the cell culture model featured here. Thus, our experimental conditions resulted in relatively constant exposure to ~0.5 µM MEHP, which is within the lower range of concentrations that have been detected in human subjects [12]. The precise kinetics of DEHP degradation in this model should be investigated in detail in future studies.

To determine whether the generation of ROS is directly dependent on DEHP as opposed to one of its metabolic products (i.e., MEHP or 2-EH), we treated all hematopoietic lineages with increasing concentrations of each of these three potential mediators. While we detected a slight and minimally concentration-dependent increase in ROS in response to the administration of either DEHP or 2-EH, both erythrocytes and dendritic cells responded to MEHP with a more substantial and clear concentration-dependent increase in ROS (Figure 3m,n). ROS levels were also higher in neutrophils in response to MEHP administration (Figure 3o). Moreover, 0.5 µM MEHP induced increases in ROS levels that were directly comparable to those detected in all DEHP-treated cultures. These findings suggest that increases in ROS observed in response to DEHP may result from the direct actions of its MEHP metabolite. Previous reports documented the role of AhR and its impact on the expression of SOD, and MEHP was identified as an AhR antagonist [68].

Thus, we examined SOD activity in each of the three cell lineages in response to treatment with DEHP and MEHP. Consistent with findings reported in previous studies, the treatment with DEHP or MEHP resulted in diminished levels of SOD; this response may contribute directly to the increases in the ROS levels observed (Figure 3p–r) [21,69,70,71,72]. Notably, the SOD activity was also reduced in neutrophil cultures treated with DEHP and MEHP, indicating an important alternative protective mechanism.

### 3.4. MEHP Induces a ROS-Mediated Shift from Glycolysis to the Pentose Phosphate Pathway, Resulting in Cell Death in Lineages without Active FAO

**Figure 4:**
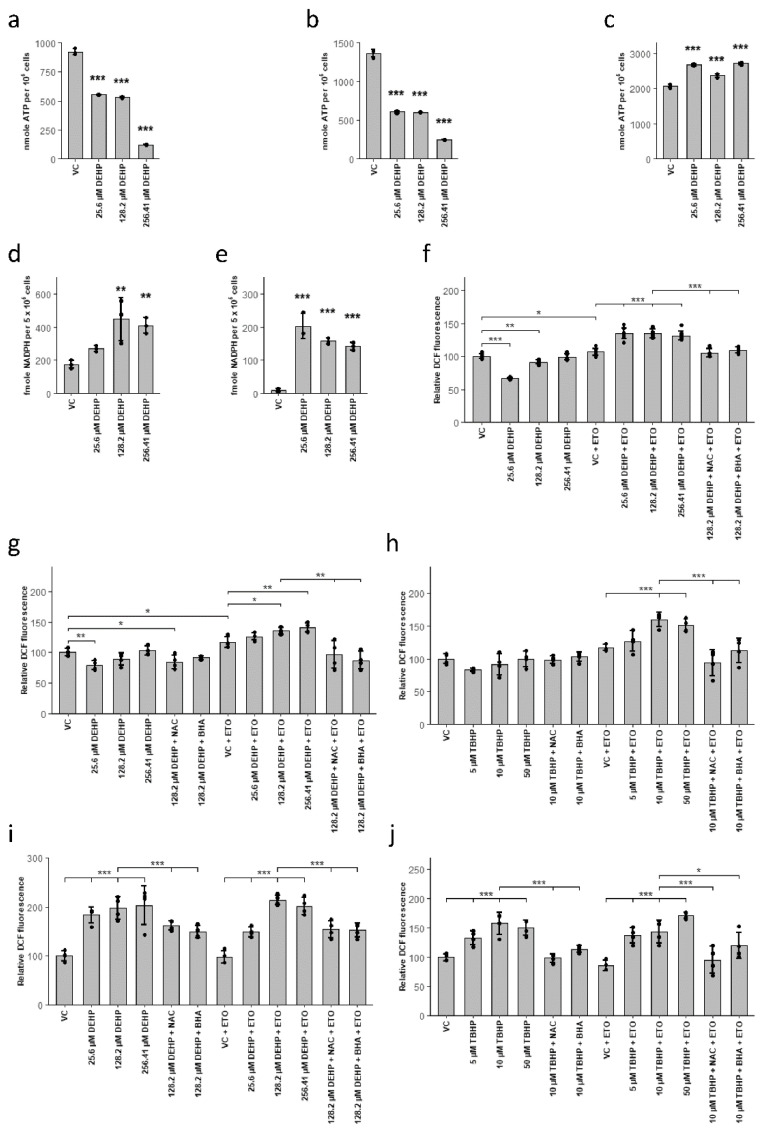
Increased oxidative stress leads to a shift from glycolysis to the pentose-phosphate pathway and ATP depletion in glycolytically active cells but not in cells capable of ATP production by fatty acid oxidation. (**a**–**c**) ATP levels after DEHP treatment of erythrocytes for 2d d (**a**), dendritic cells for 3 d (**b**), and neutrophils for 3 d (**c**). (**d**,**e**) NADPH levels after DEHP treatment of erythrocytes for 2 d (**d**) and dendritic cells for 3 d (**e**). (**f**,**g**,**i**) Relative fluorescence (DCF) in neutrophils (**f**), HepG2/C3A cells (**g**), and HUVECs (**i**) with or without preincubation with 5 µM etomoxir (ETO) and 250 µM N-acetylcysteine (NAC) or 10 µM butylated hydroxyanisole (BHA) for 2 h, followed by DEHP for 4 h (**h**,**j**). Relative fluorescence (DCF) in HepG2/C3A cells (**h**) and HUVECs (**j**) with or without preincubation with 5 µM ETO and 250 µM NAC or 10 µM BHA for 2 h, followed by tert-butyl hydroperoxide (TBHP) for 4 h. Data are presented as mean ± SEM; individual replicates are visualized as black dots in bar charts in case of *n* = 3. Statistical significance was determined by one-way ANOVA (* *p* < 0.05; ** *p* < 0.01; *** *p* < 0.001; n.s., not significant).

In addition to enzymatic inactivation (e.g., via the actions of SOD), ROS are metabolized mainly via NADPH-dependent mechanisms provided by the pentose phosphate pathway (PPP) [73]. Cells utilize a variety of different regulatory mechanisms to redirect glycolytic fluxes towards PPP to reduce ROS levels [73]. However, glycolysis is the major means of energy production in most tissues. Thus, a prolonged redirection of the glycolytic flux toward the PPP may ultimately have a negative impact on cells and tissues [74]. Glycolysis and glutaminolysis are the primary sources of metabolites that fuel the tricarboxylic acid cycle (TCA) and thus ATP production in both erythrocytes and dendritic cells [31,49,75]. Thus, we hypothesized that a prolonged redirection of these fluxes to the PPP would result in reduced ATP concentrations. We found that the administration of DEHP resulted in profound reductions in ATP levels in both erythroid and dendritic cell lineages (Figure 4a,b). By contrast, DEHP treatment resulted in increased levels of ATP in differentiating neutrophils (Figure 4c). Furthermore, DEHP treatment of erythroid and dendritic cells resulted in a considerable increase in the concentration of NADPH (Figure 4d,e), which provided further evidence for the redirection of glycolytic fluxes toward the PPP. We hypothesize that the sustained decrease in ATP levels results in decreased levels of hexokinase-mediated glucose phosphorylation which will ultimately lead to decreased substrate availability for PPP, decreased antioxidant capacity, ultimately resulting in cell death.

In neutrophils, FAO is the main source of metabolites required for the TCA and thus for the generation of ATP. This process may yield constant ATP levels and thus a stable production of NADPH via the PPP. To confirm the essential role of FAO in ROS clearance, we pre-treated differentiating neutrophils with the CPT1a inhibitor, etomoxir, immediately following DEHP treatment. Consistent with our hypothesis, the administration of DEHP resulted in significant increases in oxidative stress when FAO was inhibited (Figure 4f). Furthermore, DCF fluorescence remained at low levels upon the addition of antioxidants NAC or BHA to etomoxir pre-treated cultures. These findings confirmed that increased fluorescence was the result of higher levels of oxidative stress.

As this mechanism may be active in a general sense in cells that are capable of active FAO, we examined our hypotheses in several non-hematopoietic cells and cell lines. Among these, we treated HUVECs (which generate minimal quantities of ATP via FAO) and HepG2/C3A cells (which generate substantial amounts of ATP via FAO) with increasing concentrations of DEHP in the presence or absence of etomoxir [74,76]. Consistent with our hypothesis, HepG2/C3A cells responded to DEHP with increased ROS levels only when they were pre-treated with etomoxir (Figure 4g). By contrast, DEHP-dependent increases in ROS in HUVECs were not dependent on the actions of etomoxir (Figure 4i). Furthermore, DCF fluorescence was reduced in the presence of NAC and BHA, consistent with the observed increase in ROS. If this mechanism holds true, this relationship should not be dependent on the nature of the initial stimulus leading to an increase in ROS. Thus, we evaluated cellular responses to tert-butyl hydroperoxide (TBHP), which is another prominent inducer of ROS. Strikingly, the administration of TBHP resulted in patterns that were comparable to those observed in response to DEHP (Figure 4h,i). Collectively, these results demonstrate that the responses to DEHP are mediated via the generation of ROS and are highly dependent on FAO activity. As this relationship was also observed in other, non-hematopoietic cell lineages, it most likely represents a generalized mechanism and is characteristic of cellular behavior and function.

**Figure 5:**
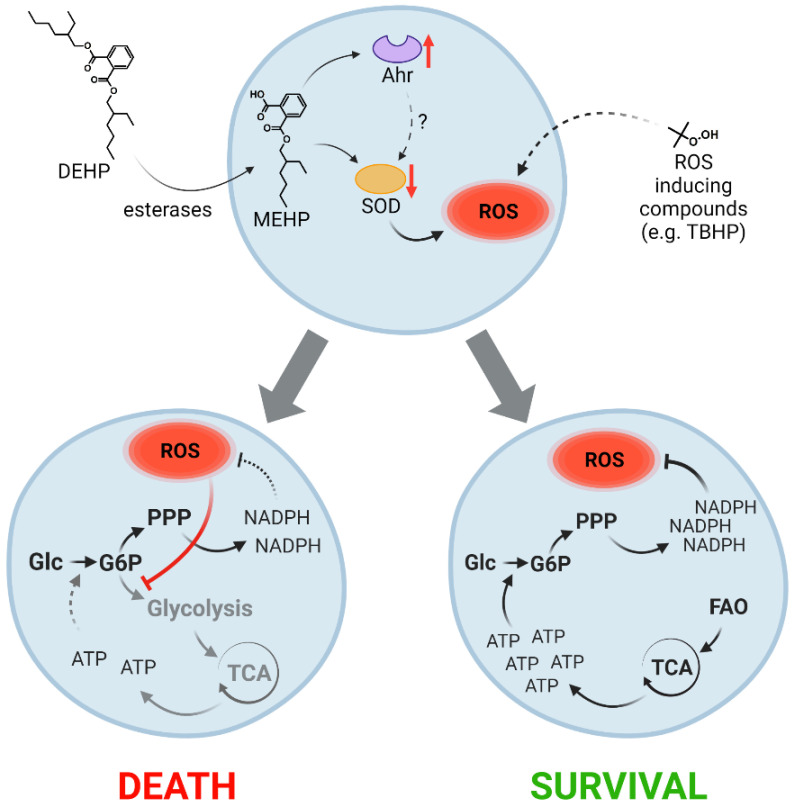
Active fatty acid oxidation increases the capacity for reactive oxygen species (ROS) quenching regardless of the nature of the stimulus. Model depicting the critical role of active fatty acid oxidation (FAO) in generating adenosine triphosphate (ATP) to support glucose (Glc) phosphorylation (to glucose-6-phosphate (G6P)), resulting in pentose phosphate pathway (PPP) activity. Administration of di-2-ethylhexyl phthalate (DEHP) results in the accumulation of ROS due to reduction of superoxide dismutase (SOD) activity mediated by its metabolite, mono-2-ethylhexyl phthalate (MEHP). The observed reduction in SOD activity may be the result of MEHP-dependent dysregulation of aryl hydrocarbon receptor (AhR) signaling. ROS can also be formed in response to unrelated mechanisms (e.g., in response to tert-butyl hydroperoxide (TBHP)). Increased concentrations of ROS redirect the glycolytic flux through PPP, leading to reduced levels of ATP (also via reduced tricarboxylic acid cycle (TCA) activity) in cells that are incapable of active FAO (left). Reduced levels of ATP result in reduced rates of glucose phosphorylation and NADPH generation, which are factors that ultimately lead to cell death. In cells capable of active FAO, ATP is generated by a mechanism that does not rely on glycolysis. These cells produce higher levels of NADPH and are thus able to quench toxic ROS (right).

Taken together, our results demonstrate that exposure to DEHP results in increased oxidative stress in all cell lineages. These responses are largely mediated by the MEHP-induced inhibition of SOD activity and may be a result of direct AhR antagonism. Increased oxidative stress that develops in response to DEHP or other stimuli promotes an increased flux to the NADPH-generating PPP. This leads to ATP depletion in cell lineages that rely on glycolysis which will ultimately result in apoptosis. However, in cell lineages capable of FAO, ATP generation is not dependent on glucose alone. These cells exhibit significantly higher resistance to the negative impact of ROS and ROS-inducing compounds (see model, Figure 5).

## 4. Discussion

Our results demonstrate that the EDC, DEHP, selectively disturbs myeloid hematopoiesis. While exposure to DEHP resulted in a profound inhibition of erythroid and dendritic cell differentiation, we found that neutrophil differentiation was enhanced. This observed increase in the rate of neutrophil maturation parallels observations made in vivo, specifically, the increased number of neutrophils detected in bronchoalveolar lavage fluid (BAL) in DEHP-exposed mice [77]. Moreover, its active metabolite, MEHP, also featured in this study, promoted decreased rates of neutrophil apoptosis and an increased production of proinflammatory proteins and chemokines [78]. Our findings provide evidence that suggests that the observed proinflammatory impact of both DEHP and its corresponding metabolite MEHP can be detected in the neutrophil lineage at earlier stages of cell differentiation.

Global metabolic profiling revealed reduced rates of glycolysis, glutaminolysis, and polyamine synthesis and enhanced apoptosis in response to DEHP treatment in both the erythroid and dendritic cell lineages. In dendritic cells (albeit not in erythrocytes), DEHP treatment appeared to inhibit total lipid anabolism. However, the total acyl-CoA pool, which controls the rate of GPL, EPC, and SL synthesis, was fuelled by cholesterol ester (CE) and triglyceride (TG) degradation in dendritic cells, while de novo lipid synthesis replenishes acyl-CoA levels in erythrocytes [31]. These different sources for lipid anabolism, in turn, may be responsible for the differential effects of DEHP on the lipidomes of these two lineages.

Collectively, our findings indicate that pathophysiologic responses to DEHP are mediated via its metabolite MEHP. MEHP induces the accumulation of ROS by inhibiting SOD, possibly via its antagonistic effects at the AhR. Of particular importance is our finding that MEHP concentrations generated in our experiments were within the range of those found in the general environment, and that inhibition of SOD could be detected in all hematopoietic cell lineages. However, despite reduced SOD activity, the administration of DEHP had no adverse effects in cultures of differentiating neutrophils. This finding provides compelling evidence for the existence of an alternative protective mechanism.

Recent studies revealed that ROS are not merely harmful metabolic by-products; they are also critical mediators of strictly regulated signaling pathways [79]. With respect to hematopoiesis and hematopoietic cell lineages, ROS are known for their critical contributions to the “oxidative burst” in phagocytic cells. ROS also promote emergency granulopoiesis and define a distinctive dendritic cell subset, thereby indicating their essential regulatory contributions to hematopoiesis [80,81,82]. Likewise, ROS quenching results in the inhibition of erythroid differentiation from progenitor cells, further underscoring the essential role of ROS and their contributions to the generation of distinct hematopoietic lineages [83]. Reductions in ROS are mainly driven by NADPH-dependent mechanisms, as NADPH promotes the reduction in both glutathione and thioredoxin [73]. The PPP is the main source of intracellular NADPH. Numerous cell types are capable of redirecting glycolytic fluxes towards PPP, thereby reducing ROS levels via one or more distinct regulatory mechanisms [73].

We also found this mechanism here, driven by ROS generation and leading to a shift in the cellular metabolism from glycolysis to the PPP. This ultimately reduces levels of ATP in cells which rely on glycolysis; this limits the use of the PPP and ultimately results in apoptosis. By contrast, ATP is generated by FAO in neutrophils; this prevents neutrophil entry into this fatal loop. This mechanism was also detected in non-hematopoietic cells and in response to other ROS-inducing compounds. Collectively, our findings suggest that this is a general mechanism that permits cells and tissues that are capable of active FAO to be more resistant to the negative sequelae of ROS.

NADPH is a major donor of reductive potential, and NADPH-generating pathways (e.g., PPP) have been recognized for their contributions to ROS-quenching. However, increased FAO activity in otherwise healthy cells and tissues is mostly recognized to cause an increase in ROS levels [84,85,86,87]. To the best of our knowledge, the connections linking the cellular responses to ROS and ROS-inducing xenobiotics to active FAO in healthy cells and tissues have received surprisingly little attention. Our findings revealed that the presence or absence of FAO activity can explain the profound differential effects of DEHP on human myeloid lineages. Thus, we recommend that future toxicological studies on these compounds might also address initial metabolic fluxes as part of their experimental design. By applying this concept, differing responses to the same stimulus observed in different cell lineages may be resolvable and lead to a better comparability of the different toxicological systems.

Comparing the concentrations of the active metabolite MEHP detected in this model (0.157 ± 0.036 µg/mL) with those reported in the general population (0.13 to 0.68 µg/mL), these results are of utmost importance. As we could demonstrate that the here observed effects are mainly caused by the metabolite MEHP at concentrations in the lower range of the frequently detected concentrations in the not highly exposed population, it is most likely that these effects can also be observed in vivo in response to DEHP release from various materials.

As DEHP is known as an immune adjuvant, and exposure is linked to the development of asthma, our findings provide a molecular mechanism that explains these observations. ROS were identified as critical for emergency granulopoiesis [80]; this finding may be directly related to the enhanced neutrophil maturation observed in our study. Increased neutrophil counts were detected in BAL fluid samples from mice in response to the administration of DEHP, providing further evidence for this mechanism in vivo [25,77]. A similar relation was found in a large multi-center retrospective study (*n* = 10,026), in which the absolute neutrophil count from serum was positively correlated with two major metabolites of DEHP [88].

In summary, our results strongly suggest that normal hematopoiesis will be disrupted in humans exposed to DEHP. Accordingly, alternative plasticizers should be identified and introduced as replacements for DEHP as soon as conceivably possible. Furthermore, our insights into the mechanisms underlying DEHP-mediated toxicity might be used when considering the impact of other ROS-generating xenobiotics. For example, in vivo administration of the ROS-inducing anti-cancer drug doxorubicin results in profound reductions in erythrocytes, lymphocytes, and monocytes, while the neutrophil lineage remains largely unaffected [89,90]. This observation may be explained at least in part by the mechanisms presented herein. Similar outcomes might also be anticipated in response to a wide range of ROS-inducing environmental chemicals, including bisphenol A, parabens, tributyltin, pesticides, and heavy metals [91]. The impact of these chemicals and materials specifically on hematopoiesis should be addressed by future studies. Moreover, the mechanisms and likely critical contribution of FAO identified in this study apply not only to hematopoiesis but likely have implications with respect to the cell metabolism in other tissues, depending on their capacity for FAO. These observations may lead to new insights and novel therapeutic approaches to the treatment of human diseases.

## Data Availability

The data presented in this study are available in the Supplementary Materials.

## Supplementary Materials

The following are available online at https://www.mdpi.com/article/10.3390/cells10102703/s1, Figure S1: Typical chromatogram obtained during separation of further sphingolipid analysis, Figure S2: The effects of DEHP are not mediated via modulation of PPARα/γ or fatty acid oxidation, Table S1: Reagents and Sources, Table S2: Analyte parameters for the applied ceramide quantification method, Table S3: Summary of concentration ranges, LOD, LLOQ, recovery rates, intra-assay CV, and inter-assay CV of the applied ceramide quantification method, Dataset S1: Significant metabolite data.
