# Supplementary material for: Lineage-Selective Disturbance of Early Human Hematopoietic Progenitor Cell Differentiation by the Commonly Used Plasticizer Di-2-ethylhexyl Phthalate via Reactive Oxygen Species: Fatty Acid Oxidation Makes the Difference
Source: Cells. 2021 Oct 9;10(10):2703. doi: 10.3390/cells10102703 (PMC8534767; doi:10.3390/cells10102703)
Supplement: Supplementary file 1 [file cells-10-02703-s001.zip › Dei_Supplement_DEHP_FAO.pdf]

Supplementary Material

Lineage-Selective Disturbance of Early Human Hematopoietic Progenitor Cell Differentiation by the Commonly Used Plasticizer di-2-ethylhexyl Phthalate via Reactive Oxygen Species: Fatty Acid Oxidation Makes the Difference

Lars Kaiser[1,2], Isabel Quint[1], René Csuk[3], Manfred Jung[2,4] and Hans-Peter Deigner[1,5,6*]

[1]Institute of Precision Medicine, Furtwangen University, Jakob-Kienzle-Straße 17, D-78054 Villingen-Schwenningen, Germany; [2]Institute of Pharmaceutical Sciences, University of Freiburg, Albertstraße 25, D-79104 Freiburg i. Br., Germany; [3]Organic Chemistry, Martin-Luther-University Halle Wittenberg, Kurt-Mothes-Str. 2, D-06120 Halle (Saale), Germany; [4]CIBSS – Centre for Integrative Biological Signalling Studies, University of Freiburg; [5]Fraunhofer Institute IZI, Leipzig, EXIM Department, Schillingallee 68, D-18057 Rostock, Germany; [6]Associated member of Tuebingen University, Faculty of Science, Auf der Morgenstelle 8, D-72076 Tübingen, Germany

*** Corresponding author:** Prof. Dr. Hans-Peter Deigner, hans-peter.deigner@hs-furtwangen.de, Phone: +49 7720 307-4232, Fax: +49 7720 307 4725, Institute of Precision Medicine, Furtwangen University, Jakob-Kienzle-Straße 17, 78054 Villingen-Schwenningen, Germany

Supplemental Table S1. Reagents and Sources.

Reagent	Supplier	Identifier
Mouse monoclonal anti-CD71-FITC	Beckton Dickinson GmbH	Cat#555536
Mouse monoclonal anti-CD235a-APC	Beckton Dickinson GmbH	Cat#561775
Mouse monoclonal anti-CD14-Pacific Blue	Beckton Dickinson GmbH	Cat#558121
Mouse IgG2ak-FITC	Beckton Dickinson GmbH	Cat#553456
Mouse IgG2bk-APC	Beckton Dickinson GmbH	Cat#555745
Mouse IgG2ak-Pacific Blue	Beckton Dickinson GmbH	Cat#558118
Mouse monoclonal anti-AhR (A-3)	Santa Cruz Biotechnology, Inc.	Cat#sc-133088
Mouse monoclonal anti-CYP1A1 (A-9)	Santa Cruz Biotechnology, Inc.	Cat#sc-393979
Mouse monoclonal anti-CYP1B1 (G-4)	Santa Cruz Biotechnology, Inc.	Cat#sc-374228
Mouse monoclonal anti-β-actin (AC-74)	Sigma-Aldrich	Cat#A2228
Donkey polyclonal anti-mouse IgG H&L-HRP	Abcam	Cat#ab6820
Human male AB serum	Sigma-Aldrich	Cat#H4522
Fetal Bovine Serum	Sigma-Aldrich	Cat#F7524
Valproic acid	Fisher Scientific	Cat#10077682
BML-210	ApexBio Technology LCC	Cat#B5968
Stemregenin 1 (SR1)	ApexBio Technology LCC	Cat#A8224
Fms-related tyrosin kinase 3 ligand (Flt-3L)	GeneScript	Cat#Z02926
Interleukin-3 (IL-3)	GeneScript	Cat#Z02991
Interleukin-6 (IL-6)	GeneScript	Cat#Z03134
Interleukin-15 (IL-15)	GeneScript	Cat#Z00377
Thrombopoietin (TPO)	GeneScript	Cat#Z03434
Stem cell factor (SCF)	GeneScript	Cat#Z02692
Erythropoietin (EPO)	GeneScript	Cat#Z02975
Etomoxir	VWR International	Cat#236020
H2DCFDA	VWR International	Cat#D6883
Troglitazone	VWR International	Cat#ALEXBML-GR210
Fenofibrate	VWR International	Cat#CAYM10005368
Etomoxir	VWR International	Cat#236020
GW9662	VWR International	Cat#ALEXBML-GR234
GW6471	VWR International	Cat#CAYM11697
Di-2-ethylhexyl phthalate (analytical standard)	Sigma-Aldrich	Cat#36735
Phthalic acid mono-2-ethylhexyl ester	Sigma-Aldrich	Cat#796832
Mono-2-ethylhexyl phthalate-d4	Fisher Scientific GmbH	Cat#15990252
Di-2-ethylhexyl phthalate-d4	Sigma Aldrich	Cat#617180
2-Ethyl-1-hexanol	Sigma-Aldrich	Cat#538051
Phenylmethanesulfonylfluoride	Sigma-Aldrich	Cat#78830
2,3,4,5,6-Pentafluorobenzylbromide	Sigma-Aldrich	Cat#101052
B-Glucuronidase	Sigma-Aldrich	Cat#3707598001
Pefabloc SC	Sigma-Aldrich	Cat#11429868001
PhosSTOP	Sigma-Aldrich	Cat#4906845001

Ethanol (LC-MS grade)	Sigma-Aldrich	Cat#111727100
Sphingosine d17:1	Avanti Polar Lipids	Cat#860640
Sphingosine d18:1	Avanti Polar Lipids	Cat#860490
Sphinganine d18:0	Avanti Polar Lipids	Cat#960498
Sphingosine-1-Phosphate d17:1	Avanti Polar Lipids	Cat#860641
Sphingosine-1-Phosphate d18:1	Avanti Polar Lipids	Cat#860492
Sphinganine-1-Phosphate d18:0	Avanti Polar Lipids	Cat#860536
C15 Ceramide	Avanti Polar Lipids	Cat#330730
C16 Ceramide	Avanti Polar Lipids	Cat#860516
C16 Dihydroceramide	Avanti Polar Lipids	Cat#860634
C18 Ceramide	Avanti Polar Lipids	Cat#860518
C18 Dihydroceramide	Avanti Polar Lipids	Cat#860627
C20 Ceramide	Avanti Polar Lipids	Cat#860520
C20 Dihydroceramide	Santa Cruz Biotechnology	Cat#sc-210989
C24 Ceramide d17:1	Avanti Polar Lipids	Cat#860650
C24 Ceramide	Avanti Polar Lipids	Cat#860524
C24 Dihydroceramide	Avanti Polar Lipids	Cat#860628
Lysosphingomyelin d18:1	Avanti Polar Lipids	Cat#860600
Sphingomyelin d18:1/16:0	Avanti Polar Lipids	Cat#860585
Tert-butyl hydrogenperoxide	Fisher Scientific	Cat#10703571
Staurosporine	VWR International	Cat#J62837.MA
N-Acetylcysteine	VWR International	Cat#A15409
Butylhdroxyanisole	Fisher Scientific	Cat#10551291
AbsoluteIDQ p180 Kit	Biocrates Life Science AG	N/A
NucleoSpin RNA XS Kit	Macherey-Nagel GmbH&Co.KG	Cat#740902
Apo-ONE Assay	Promega	Cat#G7790
ATP Detection Assay Kit - Luminescence	VWR International	Cat#700410
NADPH Assay Kit (Colorimetric)	VWR International	Cat#ABCAAB186031
Superoxide Dismutase Activity Assay Kit (colorimetric)	Abcam	Cat#ab65354
LightCycler 480 SYBR Green I Master	Roche Diagnostics	Cat#04707516001
Human: cord blood $CD34^+$ cells, mixed donors	STEMCELL Technologies GmbH	Cat#70008.3
Human: Umbilical Vein Endothelial Cells, neonatal, pooled	Sigma-Aldrich	Cat#200P-05N
Human: HepG2/C3A	ATCC	Cat#ATCC CRL-10741
HBB primers:<br>Fwd: 5'-CTC GCT TTC TTG CTG TCC A-3´<br>Rev: 5'-CAA GGC CCT TCA TAA TAT CCC C-3´	Integrated DNA Technologies	N/A
ELANE primers:<br>Fwd: 5'-CTG CGT GGC GAA TGT AAA CG-3´<br>Rev: 5'-CGT TGA GCA AGT TTA CGG GG-3´	Integrated DNA Technologies	N/A
GPNMB primers:<br>Fwd: 5'-CTG ATC TCC GTT GGC TGC TT-3´<br>Rev: 5'-CTG ACC ACA TTC CCA GGA CT-3´	Integrated DNA Technologies	N/A
RPLP0 primers:<br>Fwd: 5'-TGGCAATCCCTGACGCACCG-3'<br>Rev: 5'-TGCCCATCAGCACCACAGCC-3'	Integrated DNA Technologies	N/A
ACE 3 C8, 50x2.1mm, Ultra-Inert HPLC Column	MZ-Analysetechnik	Cat#ACE-112-0502
ACE 3 C8 Ultra-Inert HPLC Guard cartridge	MZ-Analysetechnik	Cat#ACE-112-0102GD
Rix-5Sil MS, 30 m L x 0.25 mm i.d., 0.25 µm film thickness	Restek GmbH	Cat#13623

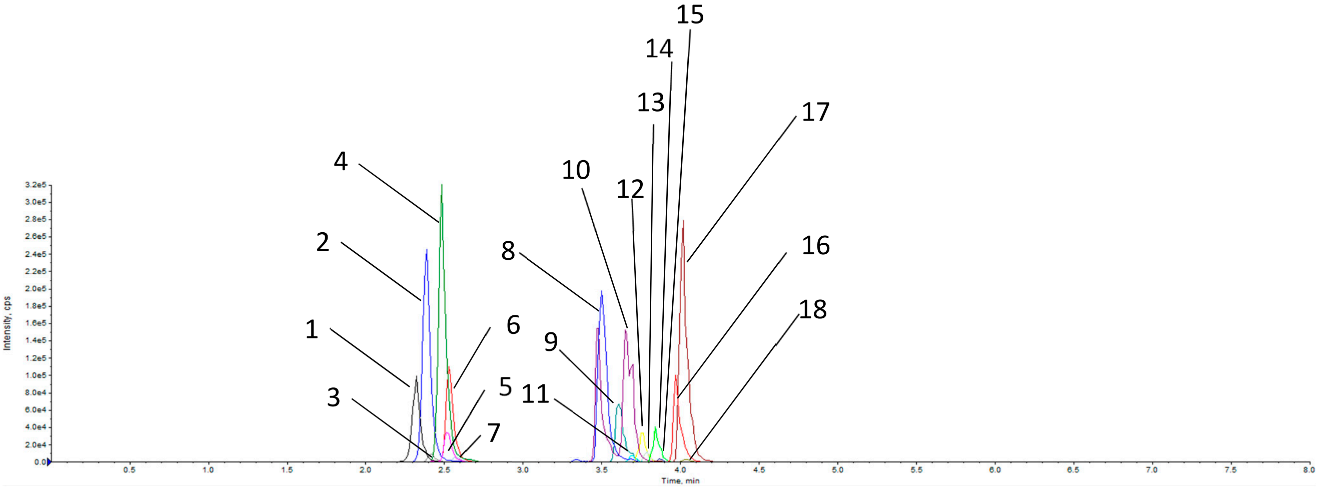

Supplemental Figure S1. Typical chromatogram obtained during separation of further sphingolipid analysis. Peaks correspond to the following analytes: **1** - lysoSM (d18:1), **2** -Sphingosine (d17:1), **3** - Sphingosine-1-Phosphate (d17:1), **4** - Sphingosine (d18:1), **5** - Sphingosine-1-Phosphate (d18:1), **6** - Sphinganine (d18:0), **7** - Sphinganine-1-Phosphate (d18:0), **8** - Sphingomyelin (d18:1/16:0), **9** - C15 Ceramide, **10** - C16 Ceramide, **11** - C16 Dihydroceramide, **12** - C18 Ceramide, **13** - C18 Dihydroceramide, **14** - C20 Ceramide, **15** - C20 Dihydroceramide, **16** - C24 Ceramide (d17:1), **17** - C24 Ceramide, **18** - C24 Dihydroceramide.

Supplemental Table S2. Analyte parameters for the applied ceramide quantification method.

Analyte	Retention time	m/z pair	Internal Standard	DP	CE	CXP
Sphingosine	2.5	300.5/282.3	Sphingosine(d17:1)	41	19	6
Sphinganine	2.54	302.6/284.5	Sphingosine(d17:1)	76	23	24
Sphingosine-1-Phosphate	2.53	380.3/264.4	Sphingosine-1-Phosphate(d17:1)	71	21	18
Sphinganine-1-Phosphate	2.59	382.3/284.5	Sphingosine-1-Phosphate(d17:1)	50	22	25
C16 Ceramide	3.67	538.4/264.1	C15 Ceramide	70	30	15
C16 Dihydroceramide	3.70	540.9/284.5	C15 Ceramide	126	47	12
C18 Ceramide	3.76	567/264.4	C15 Ceramide	40	40	15
C18 Dihydroceramide	3.79	569/284.5	C15 Ceramide	70	45	14
C20 Ceramide	3.85	595.3/264.2	C24 Ceramide (d17:1)	70	45	15
C20 Dihydroceramide	3.88	597.3/284.5	C24 Ceramide (d17:1)	121	47	16
C24 Ceramide	4.02	650.8/264.2	C24 Ceramide (d17:1)	86	43	14
C24 Dihydroceramide	4.04	653.1/284.6	C24 Ceramide (d17:1)	126	45	16
Sphingomyelin (d18:1/16:0)	3.52	704/184.2	None	150	12	15
LysoSM (d18:1)	2.33	465.5/184.2	None	56	41	20

Supplementary Table S3. Summary of concentration ranges, LOD, LLOQ, recovery rates, intra-assay CV and inter-assay CV of the applied ceramide quantification method.

Analyte	Concentration range (nM)	R^2 (extracted calibrators)	LOD (S/N > 3)	LLOQ (S/N >10)	Recovery (standard)	Recovery (cell extract)	Intra-assay CV	Inter-Assay CV
Sphingosine	1.67 - 835	0.97	3.34	3.34	78,57%	84,07%	18.15%	15.31%
Sphinganine	1.66 - 829	0.98	3.32	3.32	79,26%	88,09%	13.14%	14.04%
Sphingosine-1-Phosphate	2.64 – 1318	0.97	2.64	2.64	101,73%	73,39%	14.83%	6.09%
Sphinganine-1-Phosphate	13.1 – 1311	0.99	13.1	13.1	83,43%	63,16%	12.62%	4.86%
C16 Ceramide	1.86 – 930	0.98	1.86	1.86	96,26%	76,12%	12.00%	22.57%
C16 Dihydroceramide	1.85 – 926	0.96	1.85	1.85	103,16%	19,09%	17.35%	14.71%
C18 Ceramide	1.77 – 883	0.98	1.77	1.77	134,63%	88,7%	16.41%	17.03%
C18 Dihydroceramide	1.76 – 880	0.97	1.76	1.76	119,98%	110,38%	14.76%	6.57%
C20 Ceramide	1.68 – 842	0.94	1.68	1.68	109,62%	83,98%	20.02%	30.56%
C20 Dihydroceramide	1.68 – 839	0.93	1.68	10	97,17%	107,41%	22.31%	10.85%
C24 Ceramide	6.15 – 3076	0.95	6.15	6.15	71,17%	61,19%	16.86%	6.98%
C24 Dihydroceramide	1.53 – 767	0.98	1.53	30.76	66,1%	51,95%	30.45%	37.67%
Sphingomyelin (d18:1/16:0)	284.48 – 142242	0.97	284.48	284.48	84,14%	135,42%	13.01%	15.45%
LysoSM (d18:1)	2.15 - 1076	0.99	2.15	2.15	86,99%	91,02%	13.58%	27.56%

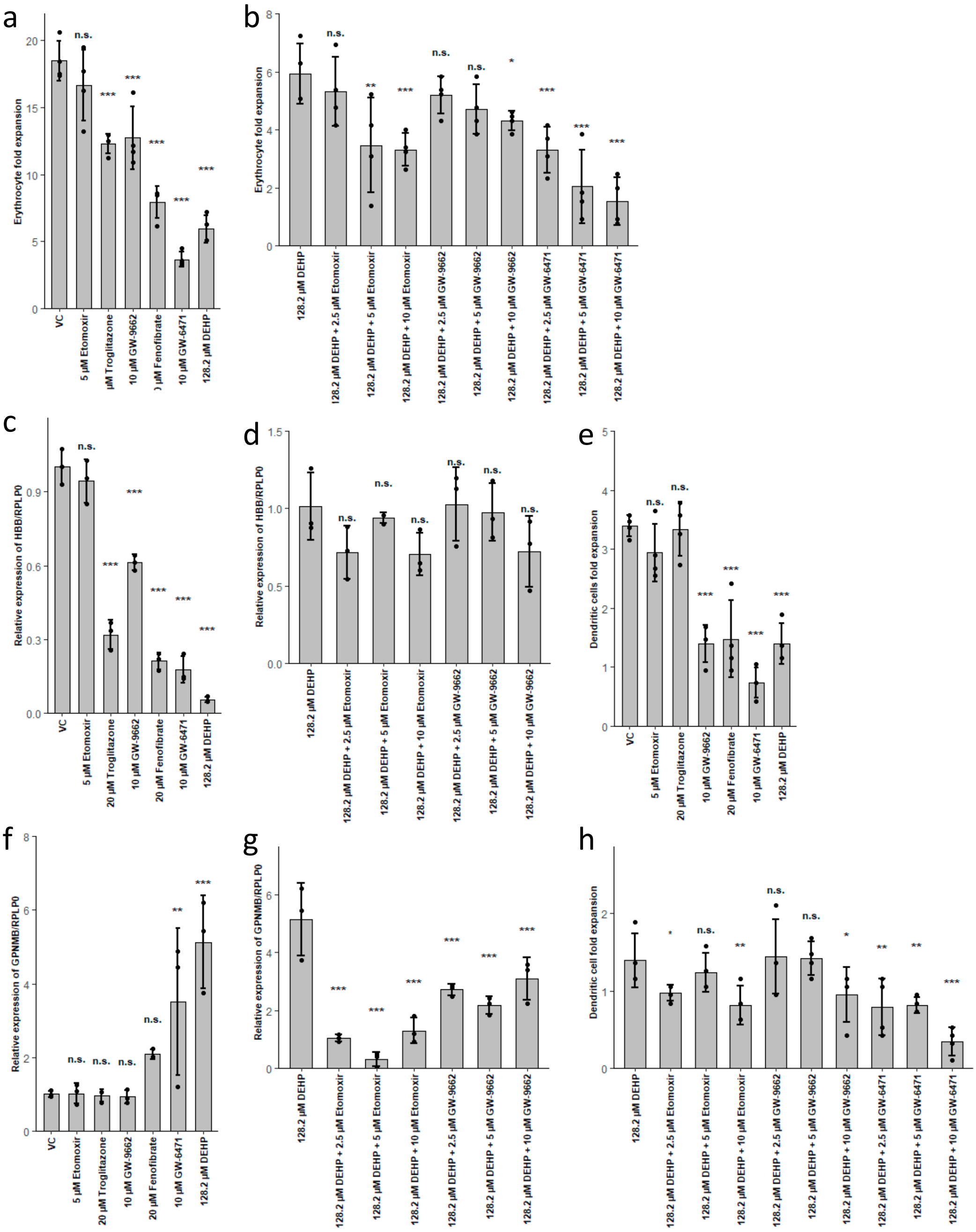

Supplemental Figure S2. The effects of DEHP are not mediated via modulation of PPARα/γ or fatty acid oxidation. (a) Expansion of erythrocytes, treated with etomoxir, troglitazone, GW-9662, Fenofibrate, GW-6471 or DEHP.(b) Expansion of erythrocytes in presence of DEHP alone, or DEHP in combination with different concentrations of etomoxir, GW-9662 or GW-6471.(c) HBB/RPLP0 expression of erythrocytes, treated with etomoxir, troglitazone, GW-9662, Fenofibrate, GW-6471 or DEHP.(d) HBB/RPLP0 expression of erythrocytes in presence of DEHP alone, or DEHP in combination with different concentrations of etomoxir or GW-9662. From samples, treated with DEHP and GW-6471, no total RNA could be extracted.(e) Expansion of dendritic cells, treated with etomoxir, troglitazone, GW-9662, Fenofibrate, GW-6471 or DEHP.(f) GPNMB/RPLP0 expression of dendritic cells, treated with etomoxir, troglitazone, GW-9662, Fenofibrate, GW-6471 or DEHP.(g) GPNMB/RPLP0 expression of dendritic cells in presence of DEHP alone, or DEHP in combination with different concentrations of etomoxir or GW-9662. From samples, treated with DEHP and GW-6471, no total RNA could be extracted.(h) Expansion of dendritic cells in presence of DEHP alone, or DEHP in combination with different concentrations of etomoxir, GW-9662 or GW-6471.
